# Neuroinflammation and Hypothalamo-Pituitary Dysfunction: Focus of Traumatic Brain Injury

**DOI:** 10.3390/ijms22052686

**Published:** 2021-03-07

**Authors:** Chiara Mele, Valeria Pingue, Marina Caputo, Marco Zavattaro, Loredana Pagano, Flavia Prodam, Antonio Nardone, Gianluca Aimaretti, Paolo Marzullo

**Affiliations:** 1 Department of Clinical-Surgical, Diagnostic and Pediatric Sciences, University of Pavia, 27100 Pavia, Italy; antonio.nardone@icsmaugeri.it; 2 Neurorehabilitation and Spinal Unit, Istituti Clinici Scientifici Maugeri SPA SB, Institute of Pavia, IRCCS, 27100 Pavia, Italy; valeria.pingue@icsmaugeri.it; 3 Department of Health Sciences, University of Piemonte Orientale, 28100 Novara, Italy; marina.caputo@uniupo.it (M.C.); flavia.prodam@med.uniupo.it (F.P.); 4 Division of Endocrinology, University Hospital “Maggiore della Carità”, 28100 Novara, Italy; marco.zavattaro@med.uniupo.it (M.Z.); gianluca.aimaretti@med.uniupo.it (G.A.); paolo.marzullo@med.uniupo.it (P.M.); 5 Division of Endocrinology, Diabetology and Metabolism, Department of Medical Sciences, University of Turin, 10124 Turin, Italy; loredana.pagano@med.uniupo.it; 6 Department of Translational Medicine, University of Piemonte Orientale, 28100 Novara, Italy; 7 Istituto Auxologico Italiano, IRCCS, Division of General Medicine, S. Giuseppe Hospital, Piancavallo, 28824 Oggebbio (VB), Italy

**Keywords:** traumatic brain injury, hypopituitarism, neuroinflammation

## Abstract

The incidence of traumatic brain injury (TBI) has increased over the last years with an important impact on public health. Many preclinical and clinical studies identified multiple and heterogeneous TBI-related pathophysiological mechanisms that are responsible for functional, cognitive, and behavioral alterations. Recent evidence has suggested that post-TBI neuroinflammation is responsible for several long-term clinical consequences, including hypopituitarism. This review aims to summarize current evidence on TBI-induced neuroinflammation and its potential role in determining hypothalamic-pituitary dysfunctions.

## 1. Introduction

Traumatic brain injury (TBI) is defined as the consequence of an external impact force, which is able to induce a transient or permanent damage of the structure and function of the central nervous system (CNS) [1,2]. TBI can be sustained by multiple and heterogeneous pathophysiological mechanisms that are responsible for complex functional, cognitive, and behavioral alterations [2]. The mechanical injury of brain tissue can originate from contusion, hemorrhage, hypoxia, and/or direct axonal injury [1,3]. The primary damage initiates a cascade of biochemical, metabolic, and inflammatory alterations leading to secondary injury, which is associated with glutamatergic excitotoxicity, vascular dysfunction, calcium overload, and neuroinflammation [4,5,6,7,8]. In recent studies, the key intermediary role of the immune system and neuroinflammation has been proposed to explain TBI pathophysiology, both in acute and long-term conditions [2,9,10,11,12]. In fact, neuroinflammatory processes can persist for several months, thereby contributing to chronic TBI alterations and accelerated brain aging in post-TBI patients [13,14,15].

The incidence of TBI has been increasing worldwide over the last few decades, reaching an incidence rate between 134 and 618 persons per 100,000 per year in different countries [16]. TBI represents the most frequent cause of death and disability in adult men in Europe [17]. Falls as well as motor vehicles and work-related accidents account for the most frequent causes of TBI [18]. The Glasgow Coma Scale (GCS) is used to classify TBI according to the level of consciousness into three categories: mild (score between 13 and 15), moderate (score between 9 and 12), and severe (score equal to or less than 8) [1]. A correlation exists between long-term clinical consequences of TBI and post-traumatic GCS severity classification [19,20,21].

Clinical consequences of TBI are heterogeneous and include both physical and cognitive disorders [22]. Depending on exogenous and endogenous factors associated with the TBI (i.e., entity and dynamics, involved anatomical sites, patient’s characteristics and physical/fitness, neurorehabilitation, and recovery outcomes), its effects may impair the function of brain areas responsible for neuroendocrine homeostasis partaking in the stress response, metabolic regulation, reproductive function, tissue trophism, and physical health. The pituitary gland plays a central role in orchestrating, along with the hypothalamus, the complex regulatory function of peripheral glands through synthesis/secretion of adenohypophyseal hormones (FSH and LH, TSH, GH, PRL, ACTH) and excretion of neurohypophyseal hormones (vasopressin and oxytocin). Alterations in pituitary functions can affect this hierarchical organization and generate global consequences in the short and long term. Hypopituitarism, the condition of pituitary hormone deficiency, results from impaired production of one or more anterior trophic hormones. Reduced pituitary function can originate from inherited disorders or, more commonly, from the damage generated by acquired conditions such as newly developing tumors or vascular, inflammatory, and infectious disorders. These processes may also impair synthesis or secretion of hypothalamic hormones, with resultant pituitary failure [23]. 

Partial or total loss of pituitary function is a known consequence of TBI. Several studies clarified epidemiology, causes, and consequences relating to post-TBI neuroendocrine disorders, conveying key information on the contribution of hypothalamic-pituitary dysfunction following mild-to-severe TBI on post-TBI morbidity and mortality [24,25,26,27,28,29,30,31,32,33,34,35]. The prevalence of post-TBI hypopituitarism is estimated to be 15–68% and progressively increases among the three categories of TBI severity described above [32,36,37,38]. Somatotropic axis deficiency represents the most frequent alteration, followed by gonadotropic, thyrotropic, and corticotropic axis deficiencies and diabetes insipidus [26,39,40]. In most cases, post-TBI hypopituitarism is transient with complete recovery within 1 to 3 years, whereas in some patients, pituitary dysfunction persists chronically or appears years after TBI [41].

The factors hypothesized to be implicated in the onset and progression/regression of post-TBI hypopituitarism include a direct mechanical injury or a vascular/hypoxic insult to the hypothalamus and/or pituitary gland and/or pituitary stalk, an increase in intracranial pressure, and alterations associated with secondary post-TBI injury [35,42,43,44]. Recently, persistent neuroinflammation has been reckoned as a potential determinant in the pathogenesis of post-TBI pituitary dysfunction [45,46,47]. The aim of the present review is to update the current data regarding TBI-related neuroinflammation and its potential role in determining hypothalamic-pituitary dysfunction.

## 2. CNS Immunometabolism, Neuroinflammation and TBI

The homeostasis of the CNS strictly depends on physiological mechanisms that are able to curb immune-mediated damage and loss of neuronal function. Encased in a rigid skull, the brain is unable to tolerate inflammation-related edema, which represents a potentially life-threatening condition [48]. The blood–brain barrier (BBB) is one of the strategies developed to finely tune the intrathecal inflammatory responses and to protect cerebral tissue. BBB is a highly selective barrier comprising the cerebral microvascular endothelium, which constitutes the interface between the peripheral circulation and the CNS, together with astrocytes, pericytes, microglia, neurons, and extracellular matrix [49,50].

In several neurological inflammatory diseases, BBB breakdown and dysfunction lead to leakage of harmful blood components into the CNS, immune cell infiltration, and aberrant passage and clearance of several molecules, contributing to neurological deficits [50,51].

**Figure 1:**
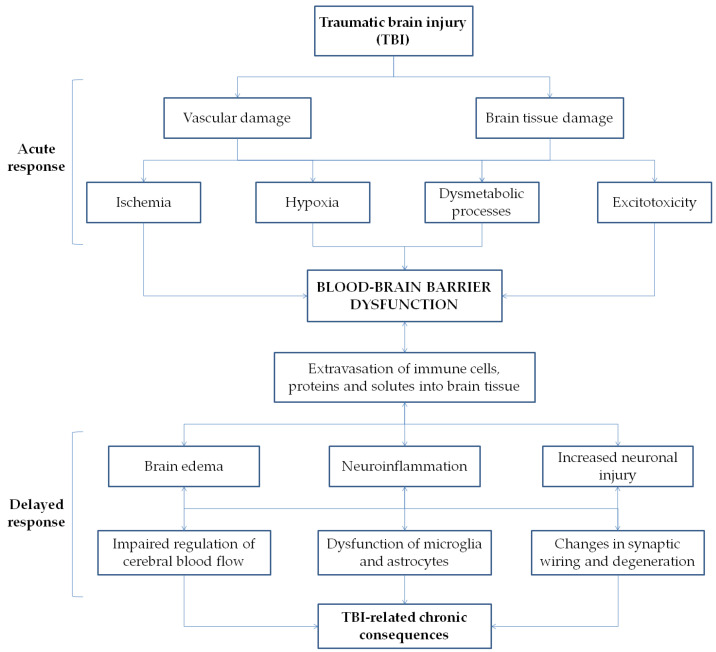
Pathophysiological events in traumatic brain injury (TBI). The primary direct injury includes vascular and brain tissue damage, associated with ischemia, hypoxia, dysmetabolic processes, and neuronal excitotoxicity. The dysfunction of the blood–brain barrier resulting from direct damage leads to extravasation of immune cells, protein, and solute into brain tissue promoting further BBB damage, cerebral edema, neuroinflammation, and neuronal damage. These mechanisms trigger a cascade of subsequent events including impaired regulation of cerebral blood flow, dysfunction of microglia and astrocytes, changes in synaptic wiring, and neuronal degeneration, which perpetuate neuroinflammation and brain tissue damage.

In the context of TBI, the development of BBB dysfunction appears to be biphasic [51,52,53] and occurs both as a direct result of the primary BBB injury and as a consequence of sustained inflammatory and cellular responses originating from the primary injury (Figure 1) [54,55,56]. The primary direct injury to BBB endothelial cells results in loosening of tight junctions with the subsequent barrier disruption [57,58]. Other mechanisms can further contribute to the local damage of BBB structural integrity, such as vasospasm, impairment of blood flow, and dysmetabolic processes [52,56]. The increased transcellular permeability of the BBB allows for the extravasation of immune cells, proteins, and solutes from the cerebral vasculature toward the interstitial space, thereby promoting edema formation, perpetuating the inflammatory response, and causing further neuronal injury [59,60]. These processes trigger a cascade of complex events directly leading to the acute complications of TBI, such as increased intracranial pressure, severe ischemic cell damage, seizure, and death [52].

Cellular membrane disruption associated with the primary mechanical injury causes the release of damage associated molecular patterns (DAMPs) such as DNA and RNA, high mobility group box 1 (HMGB1), S-100 proteins, adenosine triphosphate, uric acid, lysophospholipids, and lipid peroxidation-derived carbonyl adducts of proteins [61,62]. Through their binding to the Pattern Recognition Receptors (PRRs) on myeloid and dendritic cells, these molecules initiate the complex cascade of mechanisms that lead to post-traumatic neuroinflammation [61,63,64]. This TBI-related inflammatory response begins within hours after the injury as a part of the endogenous repairing mechanisms that should promote function restoration and lasts up to several months [65]. 

Post-TBI neuroinflammation can be described as an intricate interaction between cells of innate and adaptive immune systems [66]. The BBB dysfunction induces a focal activation of the microglia/macrophages, which exert dual beneficial and detrimental roles after CNS injury through polarization from a classical pro-inflammatory M1-like state to an alternative anti-inflammatory M2-like state [67,68]. Classical M1-like phenotype is characterized by reactive oxygen species (ROS) generation and pro-inflammatory cytokines production, e.g., tumor necrosis factor-α (TNF-α), interleukin-1β (IL-1β), whereas M2-like phenotype includes different sub-phenotypes that reflect the functional plasticity of microglia in dealing with changes of tissue microenvironment [69,70]. Studies on mice models of TBI have demonstrated microglial polarization [71,72] and the transient up-regulation of the M2-like phenotype, which is subsequently replaced by a predominant M1- or mixed Mtransition (Mtran) phenotype, associated with increased cortical and hippocampal neurodegeneration [67]. Oppositely, inhibition of M1-like phenotype has been demonstrated to improve early functional and recovery outcomes in post-TBI mice models [73].

On the other hand, astrocytes may undergo reactive astrogliosis in response to TBI, which is characterized by morphological and functional adaptation including up-production of cytokines and chemokines that further recruit and activate immune cells [74]. Elevated concentrations of glial fibrillary acidic protein (GFAP) and other astrocyte intermediate filaments (nestin and vimentin) have been shown to be associated with the severity of cellular damage, as GFAP expression is highest in reactive astrocytes in post-TBI damaged brain tissue [75]. Moreover, YKL-40, a marker of reactive astrocytes, has been found to be significantly elevated in the CNS of adults with severe TBI [76].

Pro-inflammatory cytokines and ROS are able to induce direct neuronal damage and perpetuate the pathological activation of resident CNS immunological cells, thus further increasing BBB permeability [77,78,79]. Increased systemic and intrathecal levels of pro-inflammatory mediators, e.g., TNF-α, IL-1β, and IL-18, have been documented in post-TBI patients [52,66]. In TBI animal models, it has been demonstrated that the neutralization of IL-1β is associated with an improvement of cerebral edema, contusion volume, neurodegeneration, cognitive deficits, and overall neurological recovery [80,81,82]. Improvements in post-TBI neurological outcome have also been observed in a rat model following the administration of monoclonal antibodies against TNF-α [83].

Within the injured tissue, the inflammatory mediators are able to coordinate the recruitment, expansion, and survival of peripheral immune cells [12,54,55]. Neutrophils are the first circulating immune cells to infiltrate the CNS after TBI [84,85]. Subsequently, activated T-cells are recruited along with monocytes/macrophages into traumatically injured brain areas and reflect the involvement of the adaptive immune system [86]. Whilst CNS autoreactive T-cells are typically considered harmful in autoimmune disease such as multiple sclerosis [87], their role in post-TBI setting is not yet fully understood. Some evidence suggested that the presence of autoreactive T-cells is not necessarily associated with the development of pathological autoimmunity [88], and a T cell-dependent neuroprotective response after TBI has been documented in different models of CNS injury [89]. This represents the so-called “protective autoimmunity”, which is mediated by the production of neurotropic factors from autoreactive lymphocytes that are capable of promoting the recovery of injured neurons [90,91]. In animal studies, it was demonstrated that T-cell-deficient mice exhibited poor clinical outcomes following CNS injury as compared to T-cell-competent mice, which potentially hints at the neuroprotective function of these cells [92,93]. However, other studies did not provide any evidence that T-cell-deficient mice had better outcomes than non-deficient ones in terms of BBB dysfunction, neuroinflammation, cell death, and neurological impairment [94].

In terms of duration, disseminated and chronic inflammation can persist for months after TBI [8,95,96,97], thereby favoring the pathogenesis of several degrees of cognitive dysfunction and neurodegenerative disorders, including Alzheimer’s disease (AD) and chronic traumatic encephalopathy (CTE) [8,96,98,99]. 

The long-term consequence of this inflammatory response for the BBB depends on the degree of the injury and on brain’s ability to reorganize and re-establish homeostasis [49]. In fact, mild to moderate TBI can result in transient opening of endothelial tight junctions of BBB cells, resulting in a temporally limited influx of inflammatory molecules and cells. Conversely, severe or repetitive TBI can result in a chronic alteration of BBB function, initiating focal and systemic inflammatory processes that may last for months or years [54,55,96,100,101].

Furthermore, the leaking of CNS debris and inflammatory mediators into the periphery can promote a specific complication referred to as the systemic inflammatory response syndrome (SIRS) [55,102,103,104,105]. SIRS is characterized by a state of hyper-inflammation, which elicits stress-mediated release of cortisol and catecholamines by the hypothalamus-pituitary-adrenal (HPA) axis and the sympathetic nervous system [102,103,105]. High cortisol levels can in turn affect immune system by influencing the expression of chemokines, cytokines, and adhesion molecules as well as by inducing immune cell maturation, differentiation, and migration [106,107]. These events can further worsen the neuroinflammatory setting and neurogenesis process.

In summary, TBI encompasses a complex spectrum of injuries largely related to the immune-inflammatory response during its acute and chronic phases. The immediate primary injury is considered untreatable. Instead, the pathology of the delayed second phase of damage allows a time window in which physician may act to prevent progressive neuronal death and improve patient’s recovery. Yet, improvements in the understanding of the mechanisms underlying the long-term complications of TBI could aid the development of new management strategies and effective therapeutic interventions.

### Post-TBI BBB Dysfunction in the Hypothalamic Area

In several brain areas, particularly in periventricular areas, the BBB is structured to facilitate the passage of specific substances from systemic circulation into the CNS [108]. In these areas, the BBB is more permeable thanks to the presence of highly fenestrated capillaries and fewer tight junctions [108]. The hypothalamic-pituitary area is one of the periventricular regions that exhibit these barrier properties. Moreover, in this region, the presence of a different type of radial glial cells, termed tanycytes, has also been demonstrated in the interface between the capillaries and the cerebrospinal fluid [109]. These cells have a particular aspect, which partly reflects both the morphology of ependymal cells and that of astrocytes, differing from the latter ones by having a single basal projection directed towards the brain tissue [109]. Tanycytes have a key role in regulating the passage of several substances and are able to respond to pituitary hormone production, contributing to hormone delivery to specific anatomical sites and systemic circulation [110,111]. Post-TBI BBB dysfunction can elicit specific consequences on hypothalamic-pituitary activity. First, an increase in the permeability of tanycytes could induce an imbalanced distribution of incoming and outcoming substances resulting in edema, metabolic toxicity, and local neuroinflammation, which likely alter neuroendocrine nerve terminals and homeostasis, thus potentially compromising the hypothalamic control of pituitary function [112]. Secondly, TBI-related tight-junction alterations can modify the distal end-feet of tanycytes, that are known to regulate the secretion of hypothalamic neuropeptides [113], thus compromising the physiological functioning of the hypothalamus-pituitary axis.

## 3. Molecular Patterns: Inflammasome and Inflammaging

Neuroinflammation is characterized by a host of cellular and molecular changes within the brain. Inflammasome-mediated molecular patterns and inflammatory-related aging processes (inflammaging) act synergistically so as to perpetuate post-TBI damage and associated long term complications [114].

### 3.1. Inflammasome

The inflammasome is a multi-protein platform of cytosol that is linked to the innate immune system and allows for the activation of pro-inflammatory caspases, particularly caspase-1. This platform involves important regulators of the innate immune system and hosts inflammatory responses, also defined as nucleotide oligomerization domain (NOD)-like receptors. This family of pattern recognition receptors includes three elements, namely a sensor molecule, an adaptor protein, and an effector component. Several inflammasomes have been identified in mammals, with the NLRP3 and NLRP1 being the most extensively studied in TBI [115,116]. Within the CNS, NLRP3 is mainly located in microglia, but it has also been identified in oligodendrocytes and astrocytes, while NLRP1 and a third sensor, AIM2, are expressed in neurons [116,117,118].

The molecular mechanism/s regulating NLRP3 inflammasome activation involve NRLPs as the sensors, apoptosis-associated speck-like protein (ASC, also known as PYCARD) as the adaptor protein, and caspase-1 as the effector. The first step of the process is the inflammasome priming. This is characterized by the transcriptional upregulation of NLRPs and pro-IL-1β, and post-translational modifications of NLRPs that stabilize the signal-component. The activating stimulus induces the assembly of the complete inflammasome, which is made up of seven NLRPs inflammasomes molecules arranged in a ring structure. The multimeric complex allows for the cleavage of pro-caspase-1 into the active isomer, caspase-1, which then cleaves pro-IL-1β and pro-IL-18 into active IL-1β and IL-18, respectively [116,117,118]. These cytokines are involved in the innate immune response to infections and tissue damage by creating a pro-inflammatory environment and are related to several inflammatory diseases [118,119]. While NLRP inflammasome activity seems essential to protect the host, its excessive activation can promote a form of cell necrosis termed pyroptosis, capable of mediating the neuronal death due to membrane alterations (e.g., pore formation and loss of integrity) and osmotic swelling [119].

The inflammasome can be activated by a huge variety of ligands. The best investigated are the DAMPs and pathogen-associated molecular patterns (PAMPs). Among the currently identified DAMPs and PAMPs, the prominent components include ROS, HMGB1, extracellular matrix molecules, heat shock proteins, potassium, chloride, sodium, and calcium efflux, altered calcium signaling, extracellular ATP, lysosomal destabilization, and product of mitochondrial dysfunction [70,118]. 

Within the CNS, inflammasome platforms are mainly expressed by astrocytes, microglia, and macrophages, and are activated by TBI-related neuroinflammation [70]. Animal studies showed that tissue or circulating inflammasome markers of both priming and activation processes (NLRP3, ASC, pro-caspase-1 mRNA and protein, caspase-1, IL1β, and IL18) are upregulated 6 h after a brain injury and remain elevated for more than seven days afterword [115,120]. Although studies conducted in humans are limited, they reported similar results in the blood or cerebral spinal fluid [115,121,122]. 

This pilot evidence suggests that the inflammasome machinery can act as a potential biomarker of TBI damage and could partly predict neuroinflammatory-related consequences.

### 3.2. Inflammaging

Several studies demonstrated that patients with TBI can develop long-term behavioral alterations, cognitive dysfunctions, and neurodegenerative diseases, including parkinsonisms and accumulation of the amyloid-β (Aβ) [13,14,15,123]. Recently, Fann et al. conducted a nationwide population-based observational cohort study with the aim of evaluating long-term outcome of TBI individuals [124]. These authors observed that TBI was associated with a significant increased risk of dementia as compared to subjects without TBI or suffering from non-TBI trauma [124].

**Figure 2:**
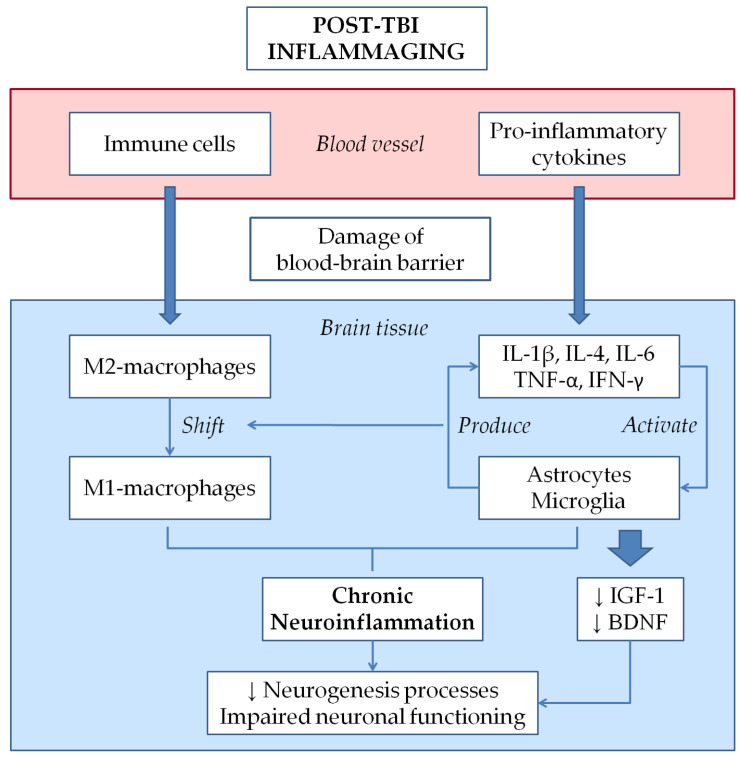
Mechanism of post-TBI inflammaging. Vascular damage caused by inflammatory molecules leads to extravasation of immune cells and pro-inflammatory cytokines into the brain tissue. These components are able to activate astrocytes and microglia, which produce further pro-inflammatory cytokines. In this setting, macrophages shift from the protective M2 phenotype towards the pro-inflammatory M1 phenotype. The consequent chronic neuroinflammation together with the decreased production of neurotrophic factors (IGF-1 and BDNF) lead to impaired neuronal functioning and neurogenesis processes.

As mentioned earlier, neuroinflammation represents a landmark of the secondary injury cascade and can often persist for months after the traumatic event, contributing to the setting of a chronic post-TBI status [2,11]. The resulting condition of chronic inflammatory post-TBI brain disease has been hypothetically linked to accelerated brain aging through a process that can be classified under the umbrella definition of inflammaging [13,14,15]. Inflammaging is characterized by a subclinical chronic inflammatory process that perpetuates neuroinflammatory post-TBI processes by modulating glial cells towards a more active pro-inflammatory state, leading to neuronal dysfunctions, loss of neuroprotective functions, and accumulation of brain tissue damage [125,126,127]. Moreover, post-TBI pro-inflammatory process associated with BBB damage, immune cell activation as well as microglia and astrocyte polarization can also contribute to decrease the production of neurotrophic factors, such as insulin-like growth factor-1 (IGF-1) and brain-derived neurotrophic factor (BDNF), which exert a key role in neuronal plasticity [125,128,129,130]. These alterations decrease neurogenesis processes and have detrimental effects for the normal neuronal homeostasis and functioning, thus contributing to an increasing risk of neurodegenerative conditions and cognitive impairment (Figure 2) [2,125].

Early evidence suggested an increased expression of the amyloid precursor protein in the acute phase of TBI, by examining post-TBI cortical brain tissue [131]. Subsequently, evidence that Aβ accumulation is accelerated by TBI has been prompted by animal and clinical studies demonstrating that TBI can acutely induce rapid Aβ production and accumulation [132,133,134].

Years after the traumatic event, changes in the perivascular matrix can still be detected in terms of increased concentration of profibrotic proteins (i.e., fibronectin and perlcan), overexpression in large blood vessels of the gatekeeper of neurological function claudin-5, as well as decreased expression of the brain-capillary transporter P-glycopreotein (P-gp) [123,135,136]. Like in the acute phase, changes in the matrix composition have been hypothesized to instigate neurodegenerative processes through Aβ accumulation and microglial activation [123,136]. The presence of Aβ plaques has been shown in 30% of TBI victims [131,137]. Post-TBI, Aβ deposits have also been found in relation to an increased expression of enzymes involved in Aβ-genesis, including the beta and gamma secretase complex proteins [138,139,140]. The synergistic action of vascular dysfunction and Aβ accumulation can activate the complement pathways around the Aβ deposits, thus perpetuating neuroinflammation and brain aging processes [2]. Similar alterations have been described in neurodegenerative CNS diseases, including AD [141]. Like in TBI, complement plays a key role in the pathogenesis of AD [142,143], where the impaired clearance of Aβ mediated by erythrocyte CR1 receptor, as well as the consequent Aβ deposition, promotes activation of the complement pathway [144]. Progressive accumulation of Aβ favors an increase in C3 complement protein, thus inducing the expression of anaphylactic C3a/C5a proteins and the formation of membrane attack complex (MAC) [145,146]. The receptors of these proteins are expressed on several CNS cell membranes including astrocytes, microglia/macrophage cells and endothelial cells. Overstimulation of such receptors and accumulation of the complement proteins are capable of damaging neurons, increasing activated glial cells and disrupting dendritic function [145,146].

All these findings could help to explain the potential role of complement in promoting post-TBI inflammaging processes [2]. As such, activation of the complement pathway has been documented in the early post-TBI stages and is possibly involved in promoting the secondary injury cascade by inducing neuronal death and alterations of the synaptic network. A complement-mediated damage could, hence, be a potential cause of long-term cognitive impairment and CNS alterations in chronic TBI [147,148,149].

## 4. The Clinical Involvement of TBI on Pituitary Functions 

The first study describing pituitary damage as a potential outcome of TBI was published in 1918 as observed in a patient with a skull base fracture showing pituitary necrosis at autopsy [150]. However, clinical awareness of hypopituitarism has expanded in the last 15 years following observations of high incidence of neuroendocrine alterations due to moderate and severe TBI [34,35,151]. Diagnosis of hormonal deficiencies is insidious, and symptoms can be non-specific and/or potentially attributed to post-traumatic stress disorder (PTSD) (i.e., fatigue, attention impairment, depression, apathy, anorexia) [152,153]. Keeping this assorted clinical context in mind, it is important to underline that delays in diagnostic processes and late initiation of appropriate replacement therapy for hypopituitarism are associated with increased morbidity and mortality [35].

Post-TBI hypopituitarism is characterized by a heterogeneous clinical spectrum that ranges from mild and non-specific symptoms to urgent conditions requiring emergency admission, including water and salt imbalance, adrenal crisis, and severe hypoglycemia [154]. Clinical manifestations depend on the number and type of pituitary axes involved, the severity of hormone deficiency, and time elapsing between hypopituitarism onset and the actual diagnosis and treatment [35]. 

Overall, growth hormone deficiency (GHD), ACTH insufficiency, and gonadotropin deficiency are the most frequent abnormalities observed in post-TBI patients [27,32,38,155]. The prevalence of these hormonal alterations varies according to the different phases of the trauma: Acute phase (1–14 days post event) and chronic phase (3–6 months post event) [156]. Each phase is characterized by specific hormonal imbalance (Table 1).

In the acute phase, ACTH-cortisol deficiency and salt/water imbalance are the most clinically relevant dysfunctions [28,153]. Growing evidence demonstrated that neuroendocrine alterations during the acute phase can be transient and likely reflect adaptative responses to post-TBI alterations [31,33,156,157,158,159]. The recovery of pituitary function has been documented, in fact, in 50% of patients with hypoadrenalism and up to 90% of patients with diabetes insipidus [156,160]. In this phase, appropriate assessment of secondary hypoadrenalism may be challenging because cortisolemia can be influenced by intrinsic factors linked to the trauma (i.e., trauma severity, presence of sepsis, therapeutic use of steroids) and the stimulatory tests may not be reliable in critically ill patients [159]. In the absence of sepsis, a morning serum cortisol ≤10 μg/dL in critical patients can be considered as inappropriately low [153,161]. In the case of central diabetes insipidus, the clinical picture is generally less confusing, and a 24 h output of 3.5 L or more of hypotonic urine in the presence of serum sodium level above the reference range can confirm the diagnosis [162]. The complete recovery occurs within days or months after traumatic event, whereas new hormone deficiency may appear in the post-acute phase [28,33].

In the chronic post-TBI phase, gonadotropin and GH deficiency are the most common pituitary alterations and are potentially responsible for chronic morbidity [163,164,165,166]. From a diagnostic viewpoint, secondary hypogonadism during the chronic phase is defined by low or inappropriately normal gonadotropins with low serum testosterone in men, low or inappropriately normal gonadotropins with low serum estradiol in premenopausal women in the absence of regular menses, and gonadotropins below the reference range for age in postmenopausal women. On the other hand, the diagnostic evaluation of the GH/IGF-I axis should be performed one year after the injury. GHD is diagnosed in the presence of impaired GH response to the GH stimulation-test (e.g., GHRH + arginine, glucagon, insulin-tolerance test), or in the presence of low-normal age- and sex-related IGF-I levels if associated with ≥3 other pituitary hormone deficits [167].

Clinically speaking, hypogonadism as well as GHD-related syndromes are characterized by decreased muscle mass and reduced bone mineral density. In addition, GHD syndrome induces peculiar metabolic and body composition alterations, including dyslipidemia, obesity, and increased visceral adiposity. Several studies demonstrated that higher BMI and abnormal lipid profile are typical of patients with post-TBI hypopituitarism and in particular if suffering from GHD, when compared to patients with normal pituitary function [168,169]. Cardiovascular alterations including premature atherosclerosis and impaired cardiac function have also been demonstrated in these patients, with a significant negative impact on quality of life (QoL) [154,169].

Along with classic endocrine symptoms of hypopituitarism, post-TBI pituitary deficiency and particularly GHD are characterized by cognitive impairment and neuropsychological complications. Although neuropsychiatric and neurobehavioral symptoms were previously considered only in the wide context of post-concussive syndrome, growing evidence demonstrated greater cognitive distress in TBI patients affected by GHD as compared to those with normal GH secretion [170], as well as more pronounced psychological distress in untreated versus treated GHD patients [169,171]. Neuropsychiatric and neurobehavioral changes range from deficit of attention, memory, information processing and execution, to more severe alterations, such as impairments in language and visuospatial constructional skills [34,170,171]. These alterations are independent of TBI severity [170]. A correlation between GH response to stimulatory tests and memory deficits has been described in patients with post-TBI GHD, as well as lower IGF-I levels have been associated to visual and memory impairment in these patients [29]. This evidence is supported experimentally by a correlation between low serum IGF-I levels and hippocampal neuron loss and spatial memory deficits [172]. 

Early identification of post-TBI hypopituitarism and a timely initiation of hormonal replacement therapy represent key elements to allow for a significant improvement of QoL and the real possibility of returning to the normal activities of daily living.

## 5. Dynamics of Post-TBI Pituitary Damage and Neuroinflammation

The mechanisms underlying post-TBI pituitary damage remain unclear. Several mechanisms have been hypothesized to exist, including the direct injury to the pituitary gland due to skull fractures, and the secondary insults relating to hypotension, hypoxia, increased intracranial pressure, changes in cerebral blood flow, and metabolism [173]. Moreover, in the last decades, a potential role of CNS inflammation in determining pituitary dysfunction has been suggested [22,47].

Given the anatomical location of the pituitary gland, both anterior and posterior gland could be susceptible to direct mechanical injury at the time of the impact. In particular, fractures through the skull base and sella turcica and the subsequent hemorrhage could directly compromise pituitary integrity and damage the pituitary stalk, leading to hypopituitarism [24,173,174,175,176,177].

The peculiar vascularization of the pituitary gland, which is characterized by long portal vessels originating from the subarachnoid space, is often compromised in TBI and is a likely pathogenetic determinant of post-TBI pituitary dysfunctions [178,179]. Shearing forces or compression due to increased intracranial pressure during TBI can damage these vessels and determine pituitary necrosis [175,176,180]. Additional secondary damage associated with hypovolemia, hypoxia, anemia, and brain swelling, which often occur as a consequence of TBI, can provide further damaging mechanisms to explain pituitary gland ischemia [173,181]. The impaired vascular-supply hypothesis could be confirmed by the anatomical pattern of hormone deficiency developing after TBI. In fact, the most frequently observed hormone defect involves GH and gonadotropins, which are indeed located in the lateral portions and pars tuberalis of the anterior pituitary gland, respectively. These areas are known to be more susceptible to ischemia due to the peculiar distribution of the portal vessels [179].

In 2009, Kasturi and Stein observed that traumatic cortical damage in male mice was associated with GHD two months after injury. The authors further demonstrated an increased concentration of GFAP and IL-1β in the hypothalamus and anterior pituitary gland [182]. Hence, they suggested that post-TBI GHD is possibly caused by local inflammatory changes and persistent astrocytosis that could involve hypothalamus and pituitary gland leading to hypopituitarism [182]. In different studies, Tanriverdi et al. observed that polymorphisms in apolipoprotein-E (APOE) are more prone to the onset of TBI-induced pituitary deficiency [43]. It is known that APO-E can downregulate the neuroinflammatory response, and it is produced in different CNS areas including the hypothalamus and the pituitary [183,184]. After the initial direct head injury, secondary neuronal damage is related to a neuroinflammatory response, which stimulates expression and release of ROS and several inflammatory cytokines such as IL-1, IL-6, and TNF [185]. APOE3 is the isoform with more pronounced anti-inflammatory properties, being able to downregulate inflammatory cytokines, both in systemic circulation and in the CNS [43,186]. Based on these findings, the authors hypothesized that TBI-induced neuroinflammatory response and the individual expression of APOE isoforms could have a key role in the pathogenesis of TBI-related pituitary damage [45]. 

In the last two decades, a potential role for TBI-induced autoimmunity has also emerged in association with hypopituitarism. Studies in mice reported on the presence of IgG autoantibodies against neuronal components after a cortical injury in adult rats and serum autoreactive antibodies against neurons in experimental TBI [187]. In 2008, Tanriverdi et al. showed for the first time the presence of serum autoantibodies against pituitary (APA) after TBI in humans [42]. Here the authors demonstrated APA in 45% of patients three years after TBI, whereas they did not find autoantibodies in the control group. A significant association between the presence of APA and TBI-induced hypopituitarism was also found [42]. Oppositely, the absence of APA was related to a significant pituitary recovery in a five-year prospective study [46]. These results led to speculate that TBI could increase the BBB permeability and cause an excessive exposure of sequestered pituitary and hypothalamic antigens [42]. It is intriguing to notice that serum APA and autoantibodies against the hypothalamus (AHA) were also assessed in a group of 61 amateur boxers, who were exposed to sport-related TBI. Serum APA and AHA concentrations were detected in 22.9% and 21.3% of subjects, respectively, and were directly associated with the onset of pituitary dysfunctions, thus strengthening the hypothesis that autoimmunity could be involved in the onset of TBI-induced hypopituitarism [45].

More recently, another mechanism has been proposed in relation to the hypothalamus-pituitary damage after TBI [22,47]. This mechanism is focused on the previously discussed tanycytes, the barrier cells of the third ventricle, which are compromised in TBI. Several studies demonstrated that tanycytes are able to regulate the hypothalamus-pituitary axes through not yet fully known mechanisms [112,113,188]. Tanycytes seem to contribute actively to the regulation of GHRH, GnRH, and TRH neuronal function, through a direct interaction with GHRH, GnRH, and TRH neuroendocrine terminals, which could be able to modulate hormonal release and pulsatility [112,188,189]. Post-TBI, altered permeability at the tanycytes barrier could represent a potential mechanism of deregulated hypothalamic-pituitary communication and, hence, function [190]. In such scenario, the pathophysiology of hypopituitarism would be predominantly caused by hypothalamic dysfunction and only secondary pituitary impairment.

In summary, neuroinflammation seems to be strongly involved in the pathogenesis of post-TBI hypopituitarism and pituitary autoimmunity could contribute to neuronal injury, intensifying the detrimental effects of neuroinflammatory patterns. An accurate identification of inflammation biomarkers and autoantibodies potentially involved in the onset and maintenance of pituitary dysfunction could allow the identification of high-risk patients and the development of therapeutic algorithms.

## 6. The Interplay between Neural Post-TBI Damage, Residual Pituitary Activity and Rehabilitation Outcomes

Short- and long-term neurophysiopathological processes act synergistically after TBI to contribute to impairment of neurological and functional outcomes [191,192] via neuroinflammation. As said earlier, peculiar central cellular and molecular patterns promote widespread neuronal damage, which compromises motor and cognitive functions [2,193,194]. From a clinical viewpoint, physical and cognitive disorders following mild to severe TBI include headache, nausea, dizziness, fatigue, sleep pattern alterations and motor dysfunctions, including loss of fine motor control and coordination, as well as difficulty with balance [193,194]. Cognitive and neuropsychological dysfunctions can be extremely disabling as they can include mental slowness, confusion, dual tasking inability, impaired memory, attention, problem solving and executive functions, which cause anxiety, irritability and depression [194,195,196].

Intriguingly, many post-TBI clinical symptoms are nonspecific and overlap with those relating to hypopituitarism [35,152]. In recent literature, post-TBI pituitary dysfunctions have been suggested to exert a detrimental effect on functional outcome at 6 months after the traumatic event, as assessed by functional independence measure (FIM) scores and mini-mental state examination (MMSE) [34]. Moreover, patients with post-TBI hypopituitarism show fatigue and a wide range of cognitive symptoms, including reduced memory performances, increased mental distress conditions, and lower scores on neuropsychological tests [197,198]. Due to this overlapping symptomatology, the diagnosis of pituitary dysfunction is often overlooked or delayed in post-TBI subjects with important consequences in terms of reduced QoL, worse neurological and functional recovery, and increased mortality [34,35,168].

As detailed earlier, GHD represents the hormonal alteration most frequently associated with both functional and cognitive impairments in post-TBI patients [163,164,165,166] and systemic consequences of GHD on lean body mass, bone mineral density, cardiac function, and cognitive impairment can negatively affect motor and functional recovery [154,169]. 

It is also worth mentioning that replacement with recombinant human GH (rhGH) in patients with GHD can significantly change the concentration of neurotransmitters in the cerebrospinal fluid (i.e., dopamine metabolite homovanillic acid and NMDA receptor ligand aspartate), thus improving cognitive functions [199,200,201,202,203,204]. However, data on the role of rhGH replacement on neurocognitive recovery after TBI are not univocal. Several studies demonstrated that rhGH replacement significantly improved cognition and QoL, while others failed to show significant effects of rhGH therapy on cognitive skills while suggesting that rhGH treatment was still able to improve fatigue and depression symptoms, which affect approximately 25–40% of post-TBI patients [29,205,206]. Of note, a recent phase II randomized, double-blind, placebo-controlled trial evaluating the efficacy of rhGH on rehabilitation outcomes from discharge after inpatient rehabilitation to the end of a 12-month follow-up failed to document significant GH-related improvements in disability scales and neuropsychological functions, while observing an improvement in FIM scores as compared to the GH-untreated group [207]. However, further studies on larger cohorts are needed to investigate the efficacy of rhGH supplementation on rehabilitation outcomes and to better define the correct timing of rhGH administration and the duration of treatment. 

## 7. Concluding Remarks

The link between neuroinflammation and neurotoxic hypothalamo-pituitary outcomes is an intriguing research area to investigate mechanisms of post-TBI pituitary damage. Immune, endothelial, and neuronal cells promote individual and synergistic responses that contribute to the impairment of pituitary homeostasis and, more in general, neuroendocrine dysfunction relating to hypothalamic-pituitary function. 

Evidence collected on the role of the inflammasome and inflammaging prompts attention on the potential role of molecular pathways aiding diagnostic workup and therapeutic approach in post-TBI hypopituitarism. Pituitary involvement after TBI has detrimental systemic effects and may negatively impact neurorehabilitation outcomes. Hence, attention is also warranted to scrutinize the role of the hypothalamic-pituitary unit on neuromotor and neurocognitive outcomes following post-TBI rehabilitation, since both neural damage and hypopituitarism have a negative influence on functional and cognitive outcomes in post-TBI patients. 

Hypopituitarism, and particularly GHD, can act centrally and peripherally through modulation of neurotransmitters (alteration of neuronal homeostasis), changes in body mass, and induction of metabolic alterations in muscle and bone. We hypothesize that these two components may, in a yet unknown number of cases, act synergistically to impair individual skills and rehabilitation outcomes. When clinical assessment and endocrine testing show abnormal responses, replacement therapy of hormone deficiencies could contribute to improve functional and cognitive skill in candidate patients’ subsets. Interventional studies are thus needed to determine the potential role for hormone replacement on rehabilitation outcomes.

## Figures and Tables

**Table 1:** Clinical features, investigative findings, and diagnostic criteria of post TBI hypopituitarism.

Hormones Deficiency	Clinical Features	Finding	Diagnosis
**ACTH**	**Acute phase:** Life-threatening adrenal crises: - Weakness, dizziness - Nausea, vomiting - Fever - Shock **Chronic phase:** - Fatigue - Pallor - Anorexia, Weight loss	- Hypoglycemia - Hypotension - Anemia - Lymphocytosis - Eosinophilia - Hyponatremia	**Acute phase:** Serum cortisol ≤10 μg/dL **Chronic phase:** - Serum cortisol ≤3 μg/dL is diagnostic - Serum cortisol ≥18 μg/dL exclude diagnosis - Serum cortisol 3–18 μg/dL consider stimulation test (corticotrophin)
**TSH**	**Chronic phase:** - Tiredness - Cold intolerance - Constipation - Hair loss, Dry skin - Hoarseness - Cognitive slowing	- Weight gain - Bradycardia - Hypotension	fT4 below the reference range with low or inappropriately normal TSH
**FSH/LH**	**Chronic phase:** Men: - Loss of libido - Impaired sexual function - Mood impairment - Loss of facial, scrotal and trunk hair - Weight changes Women: - Oligoamenorrhea - Loss of libido - Dispareunya - Infertility - Weight changes	Men: - Decreased muscle mass - Osteoporosis - Anemia Women: - Osteoporosis	Men: Low or inappropriately normal gonadotropins with low serum testosterone Women: - Low or inappropriately normal gonadotropins with low serum estradiol in premenopausal women in absence of regular menses - Gonadotropins below the reference range for age in postmenopausal women
**GH**	**Chronic phase:** - Decreased muscle strength - Visceral obesity - Fatigue - Decreased quality of life - Impairment of attention and memory	- Dyslipidemia - Premature atherosclerosis - Decreased muscle mass	- Low-normal IGF-I levels according to sex and age cutoffs associated with more than three others pituitary deficits - Impaired response to GH stimulation test (GHRH + arginine, glucagon, ITT)
**ADH**	**Acute and chronic phase:** - Polyuria - Polydipsia	- Decreased urine osmolality - Hypernatremia - Polyuria	24 h output of 3.5 L or more of hypotonic urine and serum sodium above the reference range

## Abbreviations

Aβ	Amyloid-β
AD	Alzheimer’s disease
AHA	Autoantibodies against the hypothalamus
APA	Autoantibodies against pituitary
APOE	Apolipoprotein-E
BBB	Blood–brain barrier
BDNF	Brain-derived neurotrophic factor
CNS	Central nervous system
CTE	Chronic traumatic encephalopathy
DAMPs	Damage-associated molecular patterns
FIM	Functional independence measure
GCS	Glasgow Coma Scale
GFAP	Glial fibrillary acidic protein
GHD	Growth hormone deficiency
HMGB1	High mobility group box 1
HPA	Hypothalamus-pituitary-adrenal
IGF-1	Insulin-like growth factor-1
IL	Interleukin
MAC	Membrane attack complex
MMSE	Mini-mental state examination
NOD	Nucleotide oligomerization domain
P-gp	P-glycopreotein
PAMPs	Pathogen-associated molecular patterns
PRRs	Pattern recognition receptors
PTSD	Post-traumatic stress disorder
QoL	Quality of life
rhGH	Recombinant human GH
ROS	Reactive oxygen species
SIRS	Systemic inflammatory response syndrome
TBI	Traumatic brain injury
TNF- α	Tumour necrosis factor-α
